# Evaluating of the Effects of Sub-MIC Concentrations of Gentamicin on Biofilm Formation in Clinical Isolates of *Pseudomonas aeruginosa*

**DOI:** 10.30699/IJP.20201.524220.2584

**Published:** 2021-07-06

**Authors:** Zahra Yousefpour, Fateme Davarzani, Parviz Owlia

**Affiliations:** 1 *Department of Microbiology, Faculty of Medicine. Shahed University, Tehran, Iran *; 2 *Molecular Microbiology Research Center (MMRC), Faculty of Medicine, Shahed University, Tehran, Iran *

**Keywords:** Biofilm, Pseudomonas aeruginosa, pelA, pslA

## Abstract

**Background & Objective:**

The ability of *Pseudomonas aeruginosa* to form biofilm has an important role in establishment of chronic phase of infections. Biofilm formation can be affected by antibiotics sub-MIC concentrations. The principal aim of the present study was to evaluate the effect of gentamicin at sub-MIC concentrations on biofilm formation in 100 *Pseudomonas aeruginosa* clinical isolates.

**Methods:**

Determination of minimal inhibitory concentration of gentamicin for clinical isolates was done using micro broth dilution method. The amount of biofilm formation in the treated and untreated isolates with gentamicin sub-MIC (1/2&1/4MIC) concentrations was evaluated using microtitre plate assay. *pelA *and *pslA *genes were detected in clinical isolates by PCR method.

**Results:**

99% of clinical isolates were biofilm producer. Different changes in amount of biofilm formation were observed in the treated clinical isolates with sub-MIC concentrations of gentamicin. Two dominant changes were observed in 80% of clinical isolates. These concentrations had inhibitory effect on biofilm formation in 46.4% of isolates and caused a significant decrease in its amount. While in 31.3% of the isolates, the biofilm formation was significantly increased. The frequency of *pelA *and *pslA *genes among clinical isolates was 100%.

**Conclusion:**

gentamicin sub-MIC concentrations cause different changes on biofilm formation of *Pseudomonas aeruginosa* clinical isolates. Therefore, further studies are needed for discovering new treatment strategies and using sub-MIC concentrations of the antibiotic in prevention and treatment of *Pseudomonas aeruginosa* infections.

## Introduction

*Pseudomonas aeruginosa *(*P. aeruginosa*) is an opportunistic pathogen that can form biofilms and cause nosocomial infections, especially in immunocompromised individuals (1-3). *P. aeruginosa* is known as a significant bacterium due to the ability to grow in minimal conditions, the high spread in nature and the ability to form biofilms (4). Biofilm formation is the main strategy of Pseudomonas aeruginosa to survive in harsh environmental conditions (5, 6). In the chronic phase of infections, there is a decrease in activity of most virulence factors while biofilm formation acts as a virulence factor (7). Biofilm formation reduces the effect of antimicrobial agents (8). More than 80% of bacterial infections and about 65% of nosocomial infections are related to biofilm (9, 10). The biofilm is a complex microbial community besieged by a polysaccharide or protein matrix (1, 6). Extracellular polymeric substance (EPS) or exopoly-saccharide is a viscous organic substance which is essential for the formation of biofilm structure. It consists of polysaccharides, proteins and nucleic acids. Most polysaccharides produced by *P. aeruginosa* include psl, pel and alginate. pel is a glucose cationic exopolysa-ccharide that has a major role in the establishment of solid-surface biofilms. Non-mucoid strains use pel or psl polysaccharide as the primitive constructional scaffold. The pel has a protective role in the biofilm and increases resistance to antibiotics. The *psl* gene cluster is distributed around the cell surface and strengthens the structural scaffold and improves the establishment of microcolonies. It also plays a main role in the primary attachment of sessile cells to living and non-living surfaces. The main mechanism of resistance to antibiotics and other antimicrobial factors is associated with biofilms (1). Bacterial growth in biofilm can increase the MIC of antimicrobial agents 100-1000 times (11). For a variety of reasons, the dose of antibiotic given to the patient may not achieve its optimal level (minimum inhibitory concentration, MIC or minimum bactericidal concentration, MBC) at the affected tissue place, and the bacteria expose to sub-MIC concentrations (12). Therefore, bacteria are typically exposed to sub-MIC concentrations of antimicrobial factors (13). Sub-MIC concentrations of antibiotics alter the physiological and biochemical integrity of bacteria and impair some bacterial functions (12). It can also affect bacterial virulence agents such as adhesion, cell surface hydrophobicity, biofilm formation, sensitivity to oxidative stress and motility. According to recent studies, the utilization of sub-MIC concentrations of some antibiotics, such as macrolides, may inhibit biofilm formation and be useful in the treatment of *P. aeruginosa* infections (14). On the other hand, sub-MIC concentrations of antimicrobial agents can act as a stimulus for bacterial biofilm formation (15, 16). The effect of sub-MIC concentrations of aminoglycosides on biofilm formation has been investigated in the recent years. According to some studies, sub-MIC concentrations of aminoglycosides inhibit biofilm in *P. aeruginosa *(17). Hoffman* et al.* showed that sub-MIC concentrations of gentamicin stimulate biofilm formation, while the results of Hemati* et al.*'s study showed that sub-MIC concentrations of gentamicin have no stimulatory effect on biofilm formation (13, 18). Due to different results of studies, in this research, the effect of gentamicin 1/2 MIC and 1/4 MIC concentrations investigated on biofilm formation in *P. aeruginosa* clinical isolates was.

## Material and Methods

Microorganisms and Growth Conditions

In this study, 100 clinical isolates of *P. aeruginosa* from different specimens (wound, urine, blood, etc.) were investigated. Samples collected from several selected hospitals in Tehran. Identification of clinical isolates was done by microbiological experiments including Gram staining, catalase and oxidase production, Oxidation-Fermentation (OF) test, culture on TSI medium and growth at 42°C. For final confirmation of the isolates, the *oprL* gene, which is specific to *P. aeruginosa*, was amplified by PCR and traced by electrophoresis. Clinical isolates were kept at -70°C in Nutrient broth (Merck, Germany) comprising 15% glycerol for use during the study. *P. aeruginosa *PAO1 standard strain was utilized as a positive control for biofilm assay.

Molecular Detection of *pelA*, *oprL* and *psl* Genes

PCR method was used for detecting *pelA*, *pslA* and *oprL *genes in *P. aeruginosa*. Specifications of primers are given in Table 1 (19, 20). The method is as follows: boiling method was used to extract the bacterial DNA; reaction materials (ddH2O: Primer R: 0.5 µL, Primer F: 0.5 µL, Taq DNA Polymerase Master Mix: 12.5 µL, Template DNA: 5 µL, 6.5 µL were transferred into microtubes, and for the *oprL *gene according to the protocol; Initial denaturation at 95°C for 5 min, 30 cycles included; denaturation at 95°C for 30 s, annealing at 57°C for 30 s and extension at 72°C for 1 min, and a final extension at 72°C for 10 min and for *pelA* and *pslA* genes according to the protocol; initial denaturation at 94°C for 5 min, including 34 cycles; denaturation at 94°C for 30 s, annealing at 59°C for 30 s, and extension at 72°C for 1 min and a final extension at 72°C for 10 min, it was placed in a thermocycler (BioRad-USA). Finally, PCR products were electro-phoresed in 2% agarose gel which stained with safe stain (Sinacolon, Iran).

**Table 1:** Primer sequences of *pelA*, *psl* and *oprL* genes

Reference	Product length	Primer sequence	Gene
**(** 20 **)**	656 bp	Forward: 5´-TCCCTACCTCAGCAGCAAGC -3´ Reverse: 5´- TGTTGTAGCCGTAGCGTTTCTG -3´	***pslA***
**(** 20 **)**	786 bp	Forward: 5´-CATACCTTCAGCCATCCGTTCTTC-3´ Reverse: 5´- CGCATTCGCCGCACTCAG -3´	***pelA***
**(** 19 **)**	504 bp	Forward: 5´- ATGGAAATGCTGAAATTCGGC -3´ Reverse: 5´- CTTCTTCAGCTCGACGCGACG -3´	***oprL***

MIC Determination (Minimum Inhibitory Concentration)

Minimal Inhibitory Concentration (MIC) of gentamicin for *P. aeruginosa* clinical isolates and standard strain PAO1 was determined by microbroth dilution method based on CLSI protocol (21). First, serial dilutions (0.125 - 2048 μg/mL) were prepared from gentamicin stock solution with an ultimate concentration of 5,120 μg/mL and transferred to microplate wells. Then Bacterial suspension equivalent to 0.5 McFarland standard was added to the wells. Gentamicin-free bacterial culture was used as positive control and Mueller Hinton Broth (Merck, Germany) medium was used as a negative control and were incubated for 24 h at 37°C. Then the minimum concentration of gentamicin, which inhibited bacterial growth, considered as MIC. Sensitive and resistant isolates according to CLSI (MIC ≤ 4μg/mL: sensitive، MIC ≥ 16 μg/mL: resistant) were identified. 

Biofilm Formation 

To evaluate the biofilm formation, microtiter plate method was used, with some changes (22). The Bacteria were cultured in MHB medium and incub-ation of bacteria was performed for 24 h at 37°C. Four tubes were assigned for each isolate. Then, in tube 1, the culture was diluted at a ratio of 1:100 with fresh MHB medium to give an OD of 0.5 McFarland. In tube 2, the culture was diluted at a ratio of 1:50 with fresh MHB medium to give an OD 0.2. In tubes 3 and 4, the microbial suspension of tube 2 was mixed in equal proportions with MIC and 1/2MIC concentrations of gentamicin, respectively. Therefore, in tubes 3 and 4, in the final volume, both the concentration of antibiotics and the concentration of bacteria were halved. 200 μL of the contents of each of tubes 1, 3 and 4 were transferred to microplate wells and were incubated for 24 h at 37°C. Uninoculated MHB medium was utilized as a negative control and *P. aeruginosa PAO1* standard strain was utilized as a positive control. The medium inside the wells was drained and washed 3 times with PBS (phosphate-buffered saline). To stabilize the biofilm, 200 μL of 95% ethanol was added to each well and then stained by adding 100 μL of 1% crystal violet for 5 min. To remove excess dye, washing of the wells was performed with running water. Then were placed at room temperature until dry. Finally, 150 μL of 33% acetic acid was added to the wells. The Optical Density (OD) of the wells was measured at 490 nm by ELISA Reader (PerkinElmer, USA). The experiment was done in triplicate for all 3 suspensions (contents of tubes 1, 3 and 4) related to each isolation. The mean values of three wells related to the suspension of each of tube 1, 3 and 4 were recorded as ODt and the mean OD of three wells related to the suspension of the control tube was recorded as ODc. The formed biofilm levels were determined according to the Table 2 (23).

Statistical Analysis

Data was analyzed by GraphPad Prism software version 8.0.2 using One-Way ANOVA test. P-values less than 0.05 were considered as statistically significant differences.

## Results

PCR Results

The frequency of *oprL* gene, which is specific to *P. aeruginosa* gene, as well as *pelA* and *pslA* genes that are involved in biofilm formation, was 100% in the studied isolates.

**Fig. 1:**
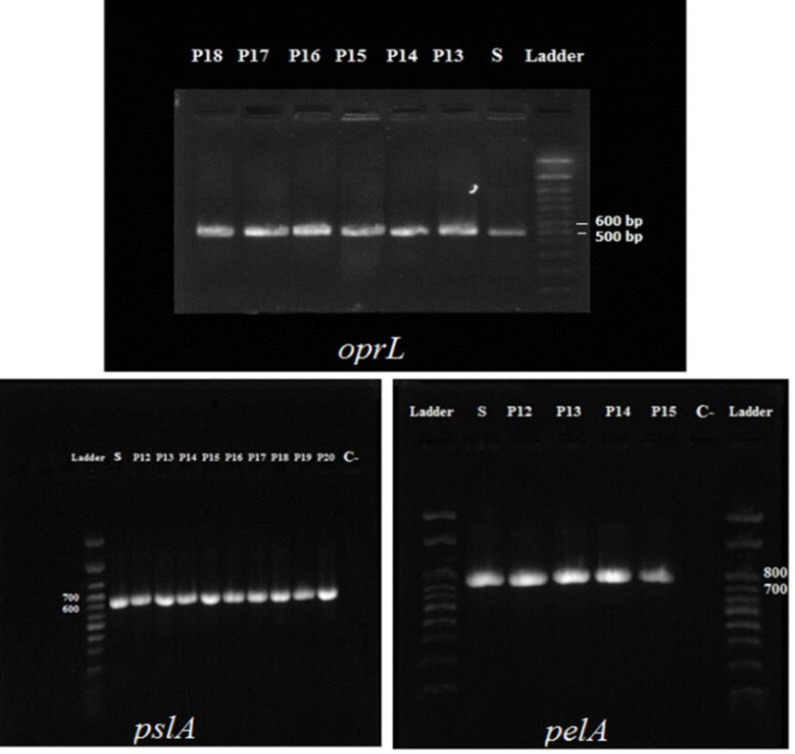
Electrophoresis of PCR products of* oprL* *(*504bp), *pelA (*786bp), *pslA* (656bp) genes. *P12- P20:* clinical isolation. S: Positive control* (*PAO1). C-: Negative control (buffer and reagent water).

Results of Electrophoresis of PCR products of *oprL*, *pelA* and *pslA* genes are given in Figure 1.

**Table 2:** Interpretation of biofilm formation

The amount of OD	Interpretation
**OD**_t_ ** ≤ OD**_c_	**Lack of biofilm formation**
**OD**_c_ ** < OD**_t_ ** < 2x OD**_c_	**Weak biofilm**
**2x OD**_c_ ** < OD**_t_ ** < 4x OD**_c_	**Intermediate biofilm**
**OD**_t_ ** ≥ 4x OD**_c_	**Strong biofilm**

**MIC Results**

MIC values of gentamicin for *P. aeruginosa *clinical isolates were evaluated according to CLSI tables. Accordingly, 71% of the strains were sensitive to gentamicin, and 29% were resistant. The frequency of MIC values of gentamicin is given in Table 3.

Effect of Sub-MIC Concentrations of Gentamicin on Biofilm Formation

**Fig. 2:**
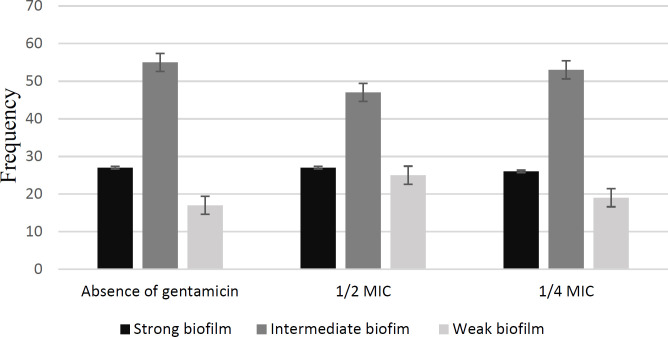
Frequency of amounts of biofilm formed in clinical isolates of *P. aeruginosa* in the presence and absence of gentamicin

**Fig 3:**
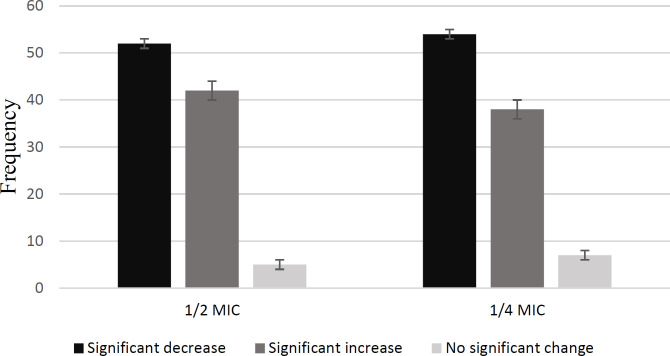
Frequency of changes in biofilm formation of clinical isolates of *P. aeruginosa*

Overall, 99% of clinical isolates were biofilm producer. The amount of biofilm formation of *P. aeruginosa* PAO1 standard strain decreased significantly in both of 1/2 MIC and 1/4 MIC concentrations and formed a strong biofilm in the absence of gentamicin. Frequency of amounts of biofilm formed in clinical isolates of *P. aeruginosa* in the presence and absence of gentamicin are given in Figure 2. Sub-MIC concentrations of gentamicin had different effects on clinical isolates of *P. aeruginosa *(Figure 3). In exposure to both 1/2 MIC and 1/4 MIC concentrations of gentamicin, 46.4% of the isolates had a significant decrease, and 31.3% had a significant increase (Table 4).

**Table 3:** Frequency of gentamicin MIC values in clinical isolates of *P. aeruginosa*

512	256	128	64	32	16	2	1	0.5	0.25	MIC(μg/mL)
6	12	5	1	4	1	3	20	37	11	**Frequency**

**Table 4:** Frequency of biofilm formation changes in clinical isolates of *P. aeruginosa* in the presence of 1/2 MIC and 1/4 MIC concentrations of gentamicin

Percentage	Frequency	Type of changes
**% 31.3**	31 Isolates	**Significant increase in 1/2 MIC and 1/4** **MIC**
**% 46.4**	46 isolates	**Significant decrease in 1/2** ** MIC and 1/4** ** MIC**
**%** ** 5**	5 isolates	**Significant increase in 1/2 MIC, Significant decrease in 1/4** ** MIC**
**%** ** 3**	3 isolates	**Significant decrease in 1/2** **MIC, significant increase in 1/4** ** MIC**
**%** ** 6**	6 isolates	**Significant increase in 1/2** ** MIC, No Significant change in 1/4 MIC**
**% ** ** 4**	4 isolates	**No Significant change in 1/2 MIC, significant increase in 1/4 MIC**
**% ** ** 3**	3 isolates	**Significant decrease in 1/2 MIC, No Significant change in 1/4 MIC**
**%** ** 1**	1 isolate	**No Significant change in 1/2 MIC and 1/4 MIC**

## Discussion

*P. aeruginosa* is an opportunistic pathogen with minimal nutritional requirements and causes various types of nosocomial infections (24). This bacterium is able to form biofilms therefore often found in patients with chronic infections (25). Biofilm formation leads to the progression of the acute phase of infections to the chronic phase and plays a significant role in *P. aeruginosa *infection (26). Therefore, removal of bacteria and its biofilm is essential for the treatment of infections (27). Biofilms are resistant to antibiotics and cleared by the host immune system and are difficult to remove by antibiotics (28, 29). According to studies, treatment with sub-MIC concentrations of antimicrobial agents is effective on biofilm formation in *P. aeruginosa* (26). Sub-MIC concentrations of some antibiotics have been reported in a number of studies as inducer and in others as inhibitors of biofilm formation. It is essential that the effect of sub-MIC concentrations of antibiotics evaluated in different clinical isolates, as bacteria are subjected to sub-MIC concentrations of antibiotics at the beginning and end of the treatment regimen, between doses, or during continuous low-dose treatment (30). In our study, the biofilm formation of 100 clinical isolates of *P. aeruginosa was* investigated in the treated and untreated state with sub-MIC concentrations of gentamicin. According to Bahador* et al.* 98.6% of clinical isolates of *P. aeruginosa* (75 isolates) from diverse samples, were able to form biofilms, which formed 60% of the isolates a strong biofilm, 34.3% a moderate biofilm and 4.3% a weak biofilm (31). Bahador* et al.* investigated the biofilm formation in antibiotic-untreated isolates, while we investigated the isolates in both of antibiotic-treated and untreated state. Based on the study of Purnajaf* et al.*, of 143 clinical isolates of *P. aeruginosa *isolated from patients with cystic fibrosis, 78.2% of the isolates formed biofilm and *pelA* and *pslA* genes were present in 57.3% and 89.5% of the isolates, sequentially (32). Ghadakaz* et al.* studied 104 clinical isolates of *P. aeruginosa*, and observed biofilm formation in 50.9% of the isolates, and the frequency of *pelA* and *pslA *genes were 45.2% and 83.7%, respectively (20). Based on the results of our study, all isolates (100%) were positive for *pelA* and *pslA* genes. Overall, 99% of the isolates formed the biofilm and only one isolate did not form a biofilm despite having the mentioned genes. Intermediate biofilm had the highest frequency in the amount of biofilm formed by isolates. So that in the absence of gentamicin and in exposure to 1/2 MIC and 1/4 MIC concentration of gentamicin, 55, 47 and 53 isolates were formed intermediate biofilm, respectively. Variances in studied isolates caused differences in the results of mentioned studies compared to the results of present study. Various studies have reported different effects of antibiotics sub-MIC concentrations on biofilm formation and other factors related to bacterial pathogenesis. Otani* et al.* evaluated the expression of *pelA* and *pslA* genes and the biofilm formation of PAO1 strain in the untreated and treated state with ceftazidime sub-MIC concentrations (1/4, 1/8 and 1/16 MIC). In their study, sub-MIC concentrations of ceftazidime reduced the expression of *pelA* and *pslA *genes by 1.8- 2.1 times and also reduced the biofilm formation of *PAO1* strain (26). According to Bala* et al.* sub-MIC concentrations of azithromycin had an inhibitory effect on biofilm formation, production of signaling molecules of quorum sensing and motility of 25 clinical isolates of *P. aeruginosa* (28). The results of our study showed various changes in the biofilm formation of clinical isolates of *P. aeruginosa* in exposure to sub-MIC concentrations of gentamicin. The two main effects of these concent-rations were inhibitory effect and stimulatory effect on biofilm formation which were observed in 46.4% and 31.3% of isolates, respectively. The results of our study in PAO1 strain and 46.4% of the isolates are in accordance with Otani and Bala study. The results of a number of studies indicate the stimulatory effect of sub-MIC concentrations of antibiotics on the biofilm formation of various bacteria. R. Hoffman* et al.* obser-ved that tobramycin sub-MIC concentrations stimula-ted biofilm formation in *P. aeruginosa PAO1* (18). This is against our results and the Otani's study on PAO1. It seems that the difference in the type of antibiotic used causes a difference in the results. Hemmati* et al.* observed that sub-MIC concentrations of ceftazidime, piperacillin, ticarcillin, carbonicillin, amikacin, ciprofloxacin, and the biocides, including chlorhexidine and benzalkonium chloride, increased the biofilm formation in 10 clinical isolates of *P. aeruginosa *(13). Based on the study of W. Garey* et al.* sub-MIC concentrations of clarithromycin increased the adhesion and volume of biofilm of 44 clinical isolates of *P. aeruginosa* (15). In the present study, in 31.3% of isolates, gentamicin sub-MIC concentrations increased biofilm formation. Majtan* et al.* found that the 1/4 MIC concentrations of the antibiotics genta-micin, amikacin, tobramycin, streptomycin, netilmicin, ciprofloxacin, norfloxacin, afloxacin and nalidixicacid, had an inhibitory effect on alginate (One of the important polysaccharides of biofilm structure in mucoid strains) production in one clinical strain of *P. aeruginosa* while 1/16 MIC concentration of some of these antibiotics had no effect on alginate and some others had a stimulatory effect on alginate production (33). We also observed that the reaction of some isolates in exposure to 1/2 MIC concentration was different from their reaction to 1/4 MIC concentration of gentamicin, in 5% of isolates. 1/2 MIC concentration of gentamicin had a stimulatory effect and 1/4 MIC concentration of gentamicin had an inhibitory effect on biofilm formation. Reverse results were observed in 3% of isolates. On the other hand, in some other isolates, only one concentration affected the biofilm formation. Thus, in 6% of isolates, 1/2 MIC concentration of gentamicin increased biofilm formation, but 1/4 MIC concentration had no significant effect on biofilm formation. In 4% of isolates, the 1/4 MIC concentration of gentamicin induced biofilm formation, while the 1/2 MIC concentration had no significant effect on biofilm formation. To confirm the different effects, the experiments were repeated and the same results were obtained again. Bruchmann* et al.* evaluated the effect of sub-MIC concentrations of several antibiotics on *P. aeruginosa* isolates collected from municipal waste-water. An increase was observed in biofilm biomass and thickness in treated state with sub-MIC concentration of sulfamethoxazole and erythromycin, in expression of resistance genes and efflux pump in the presence of sub-MIC concentrations of sulfonamides and in expression of quorum sensing genes in exposure to sub-MIC concentrations of macrolide. Sensitive isolates to these antibiotics had higher transcriptional activity (34). In the present study, the dominant change observed in both susceptible and gentamicin-resistant isolates included a reduction in biofilm formation. The inhibitory effect of sub-MIC concentrations of gentamicin was observed in 52.11% of susceptible isolates and 42.85% of resistant isolates. Based on the results of the studies, the response of bacteria to sub-MIC concentrations of antibiotics depends on the type of antibiotic, type of isolate or strain and test conditions (34). Because sub-MIC concentrations of antibiotics play a significant role in the regulation and modification of a wide range of bacterial genes, including virulence genes, and given that the expression of virulence genes and biofilm formation in *P. aeruginosa* is regulated by the quorum sensing, the changes observed in exposure to sub-MIC concentrations of antibiotics may be due to interaction with the quorum sensing system (32, 35). 

## Conclusion

The results of present study demonstrated two dominant reactions. The stimulating effect of sub-MIC concentrations of gentamicin on biofilm formation can make difficult the treatment and lead to the creation or persistence of chronic infections. The inhibitory effect of these concentrations could provide new therapeutic strategies. Due to the different results of the present and other studies, the use of sub-MIC concentrations in the treatment process requires more comprehensive studies. It seems that the study of factors involved in the different responses of bacteria to sub-MIC concen-trations of antibiotics can be helpful in achieving the desired results.
